# IL-15 in alopecia areata: the immune tolerance mechanistic insights and translational perspectives

**DOI:** 10.3389/fmed.2026.1815855

**Published:** 2026-08-24

**Authors:** Jiahua Fan, Yang Luo, Chong Gao, Yanqing Wang

**Affiliations:** 1 Department of Dermatovenereology The second Hospital of Shanxi Medical University, Taiyuan, Shanxi, China; 2 Department of Pathology, Joint Program in Transfusion Medicine, Brigham and Women’s Hospital/Children’s Hospital, Harvard Medical School, Boston, MA, United States

**Keywords:** alopecia areata, IL-15, tolerance, immunoregulation, Treg cell

## Abstract

Alopecia areata (AA) is a highly prevalent human autoimmune disease. It is now regarded by most scientists as a defect of immune privilege (IP). The impaired IP in the hair follicles is an essential driver of AA, which is developed by the imbalanced local CD8^+^ T cell, NK cell, tissue-resident Treg cells and infiltration of many factors such as IFN-*γ* and TGF-*β*. In the past, IL-2 and IL-15 are generally recognized for their roles in promoting pro-inflammatory immune responses. Moreover, cytokine-based therapeutic strategies utilizing IL-2 or IL-15 have the capacity to expand effector T cell and NK cells, thereby offering a viable alternative immunotherapeutic approach for cancer treatment. In contemporary century, there has been a gradually growing recognition of their critical role in modulating immune tolerance (via Treg cell). Low-dose IL-2 preferentially induces Treg cell, recovering the balance status between Treg cell and effector T cell and representing an original strategy for autoimmune systematic diseases. Recent study found IL-15 was the guardian of IP in hair follicles arousing the attention of IL-15 in maintain immune tolerance. IL-15, as a pleiotropic cytokine, shares many overlapping biological functions with IL-2. Notably, IL-15 will replace IL-2 to play a certain role while the level and function of IL-2 has decreased. Low-dose IL-2 and certain concentration of IL-15 complement each other, maintaining the function of Treg cell, and induce reconstruction in immune tolerance, which may be a new strategy for treating alopecia areata. Therefore, refocusing on the function of IL-15 and its role in AA might provide new insights into the pathogenesis and therapy of AA.

## Graphical abstract

**Figure:**
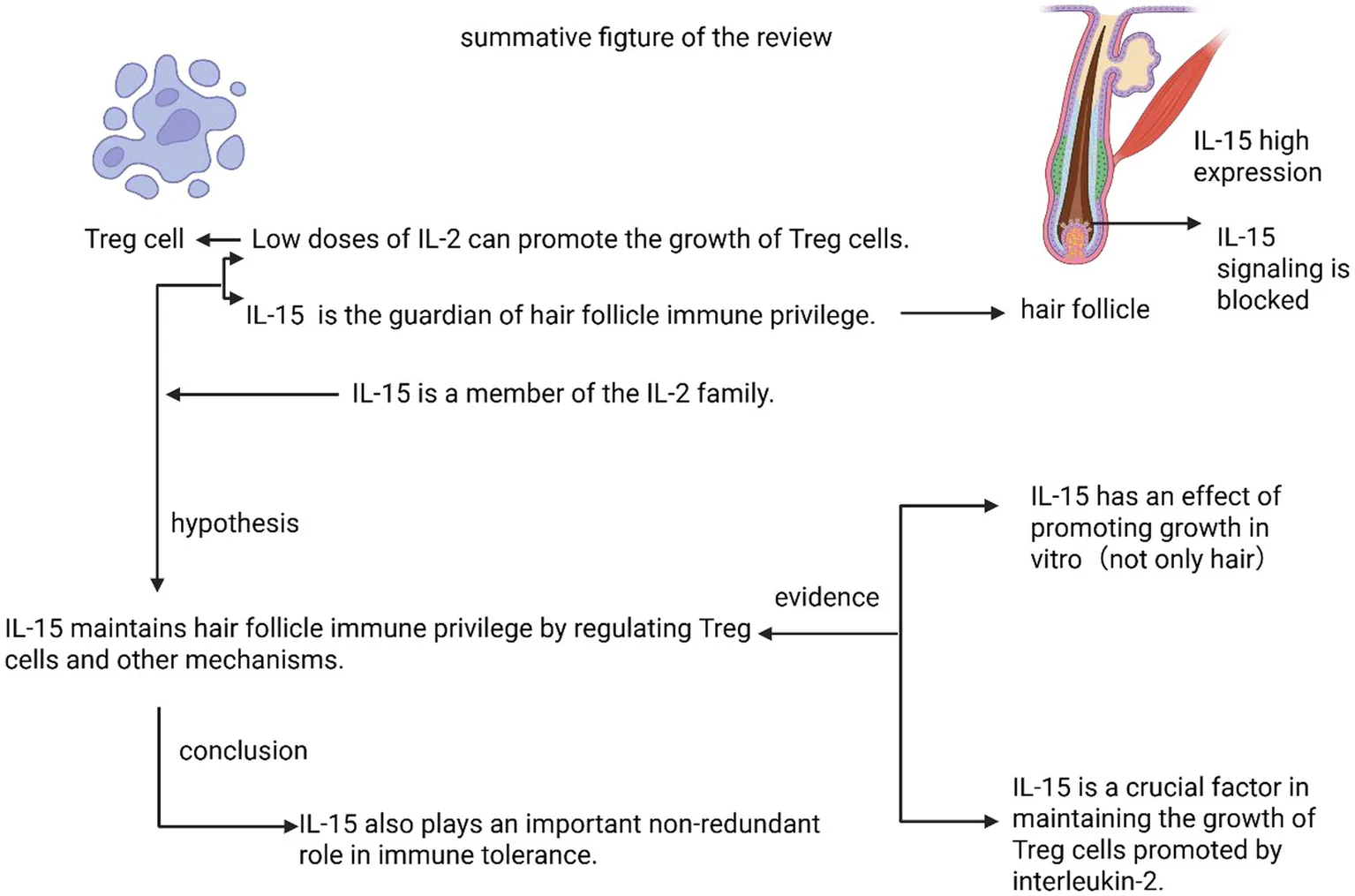
Created in BioRender. Jiahua Fan, https://BioRender.com/8o400pb.

## 1 Introduction

Alopecia areata (AA) is a kind of a non-scarring form of hair loss driven by autoimmune-mediated inflammation (1). Obviously, it ranks among most common autoimmune dermatologic conditions, affecting approximately 2% human at certain stage in their lives. Clinically, AA manifests as either patchy, localized hair loss or more extensive, diffuse hair loss (2). It is well-established that the pathogenesis of AA involves genetic predisposition, infectious agents, psychological stress, immune dysregulation and various other factors, however the specific pathogenesis is not completely clear at present. The dominant theory posits that AA arises from the inflammatory and autoimmune damage of hair follicles (3). The growth of healthy hair follicles undergoes an independent cycle including anagen, catagen and telogen phases. The hair growth cycle of AA is disrupted, mainly manifested as the affected hair follicles entering the terminal stage prematurely, while also preventing the progress of the initial stage. This process is mediated by abnormal inflammatory responses under the effects of natural killer (NK) cells, CD8^+^ T cells and cytokines such as interferon-*γ* (IFN-γ) and tumor necrosis factor-*α* (TNF-α) (4). The main target antigens of hair follicles are melanocyte-related molecules and certain structural proteins. Healthy hair follicles are protected by immune privilege (IP) through the absence of lymphatic drainage and the presence of extracellular matrix, which acts as a physical barrier, and the induction of apoptosis of immune cells capable of breaking through this barrier. Additionally, skin-resident regulatory T (Treg) cells, a special population of Treg cells preferentially localized to hair follicles, also produce cytokines that inhibit the expression of major histocompatibility antigens (MHC) to protect their hidden antigens and maintain the IP (5, 6). When IP in hair follicles is disrupted by specific triggers, the antigens of hair follicles will be exposed to the immune system to activate immune responses, which is thought to be an important driver of AA. Consequently, inhibit the immune response and reestablish the IP in hair follicles might be potential therapy for AA.

Low-dose interleukin-2 (IL-2) is an effective therapy for autoimmune diseases by inducing Treg cells (7, 8), and some promising data also have been achieved with IL-2 to treat AA (9–11). IL-2 belongs to the *γ*c family of cytokines because the receptor of IL-2 contains a γ-chain to play an important role in signal transduction. Besides IL-2, γc family of cytokines also include IL-4, IL-7, IL-9, IL-15 and IL-21. Among them, IL-15, as a pleiotropic cytokine, has gradually attracted widespread attention from researchers. On the one hand, IL-15 has been recognized as a critical regulator of memory CD8^+^ T cell, NK, NKT and γδT cell (12) to preferentially sustain the survival, proliferation and long-term maintenance of these cells. These properties make IL-15 a more favorable cytokine for therapeutic strategies aimed at promoting durable antitumor immunity. On the other hand, the receptor of IL-15 and IL-2 share IL-2Rβγ chain, which suggests that IL-15 may also play a role in the development of Treg cells. Studies have found that IL-15 could promote the formation of immune tolerance by regulating the proliferation of Treg cells and promoting growth and repair to a certain extent, which have similar effects and possible links with low dose IL-2 (13–18). Treg cell is a suppressive CD4^+^ T cell that restrain inflammation (19, 20). Measures targeting Treg cells have shown great promise in repairing damaged tissues and restoring immune tolerance for treating autoimmune diseases including AA (21–24). So far, multiple clinical trials seeking to enhance endogenous Treg cells or deliver them as a cell-based therapy have been performed and hint at signs of success, as well as to important limitations and unanswered questions (25, 26). Based on the effect of IL-15 on Treg cells, it might have broad prospects in treating autoimmune diseases (27, 28). The pleiotropic effects of IL-15 depend on its presentation mode, the concentration, the activation status of target cells and the cytokine network in the microenvironment. In a stable microenvironment, IL-15 acts as a guardian of immune tolerance, while in an inflammatory microenvironment, it transforms into a pro-inflammatory amplifier. However, the role of IL-15 in the pathogenesis of AA remains unclear. We will summarize the structure and signal transduction process of IL-15, explore its pro-inflammatory and immunoregulatory functions, and provide new insights into the pathogenesis of AA. Meanwhile, since low-dose IL-2 works synergistically with specific doses of IL-15 to promote Treg cell proliferation and induce immune tolerance and tissue repair, IL-15 may offer a new treatment option for AA, we also discuss the latest research progress about it. This is an important and timely topic regarding the research on the pathogenesis and treatment strategies of alopecia areata involving IL-15.

## 2 Biological characteristics of IL-15

### 2.1 IL-15 and IL-15 receptors

Grabstein et al. first found a certain cytokine that could maintain T cell function in the supernatant of monkey kidney epidermal cell line in 1994, and then successfully cloned the gene and named it IL-15 (29). IL-15 is a glycoprotein, which with a molecular weight of 14 ~ 15 kDa and its structure is composed of 169 amino acids (30). Human IL-15 gene is located on chromosome 4q31, with a span of about 34 kb, 7 introns and 9 exons, and its mRNA length is 1.5 ~ 1.8 kb. The isoelectric point of IL-15 is about 4.35, and it is relatively stable when the pH value is 2 ~ 10. IL-15 is engendered by a variety of immune and non-immune cells including monocytes, macrophages, dendritic cells, stromal cells, intestinal epithelial cells. Like IL-2, IL-4, IL-9 and IL-21, it also belongs to γc family of cytokines with four alpha-spiral bundle (31, 32). Although IL-15 and IL-2 are not homologous products, the receptor of them share IL-2Rγ and *β* chain except for the *α* chain (33). IL-15 receptors (IL-15R) are composed of IL-15Rα (CD215), IL-2/15Rβ (CD122), and IL-2/15Rγ (CD132) (34, 35). Therefore, IL-15 can play a biological role similar to IL-2 when the body is in the condition of IL-2-scarcity. Because of the difference of *α* receptor, IL-15 receptor distribution is also different from IL-2. The three polymerized to form a high-affinity receptor and when the *α* receptor is lacking, a low/intermediate affinity receptor is formed (36).

**Figure 1:**
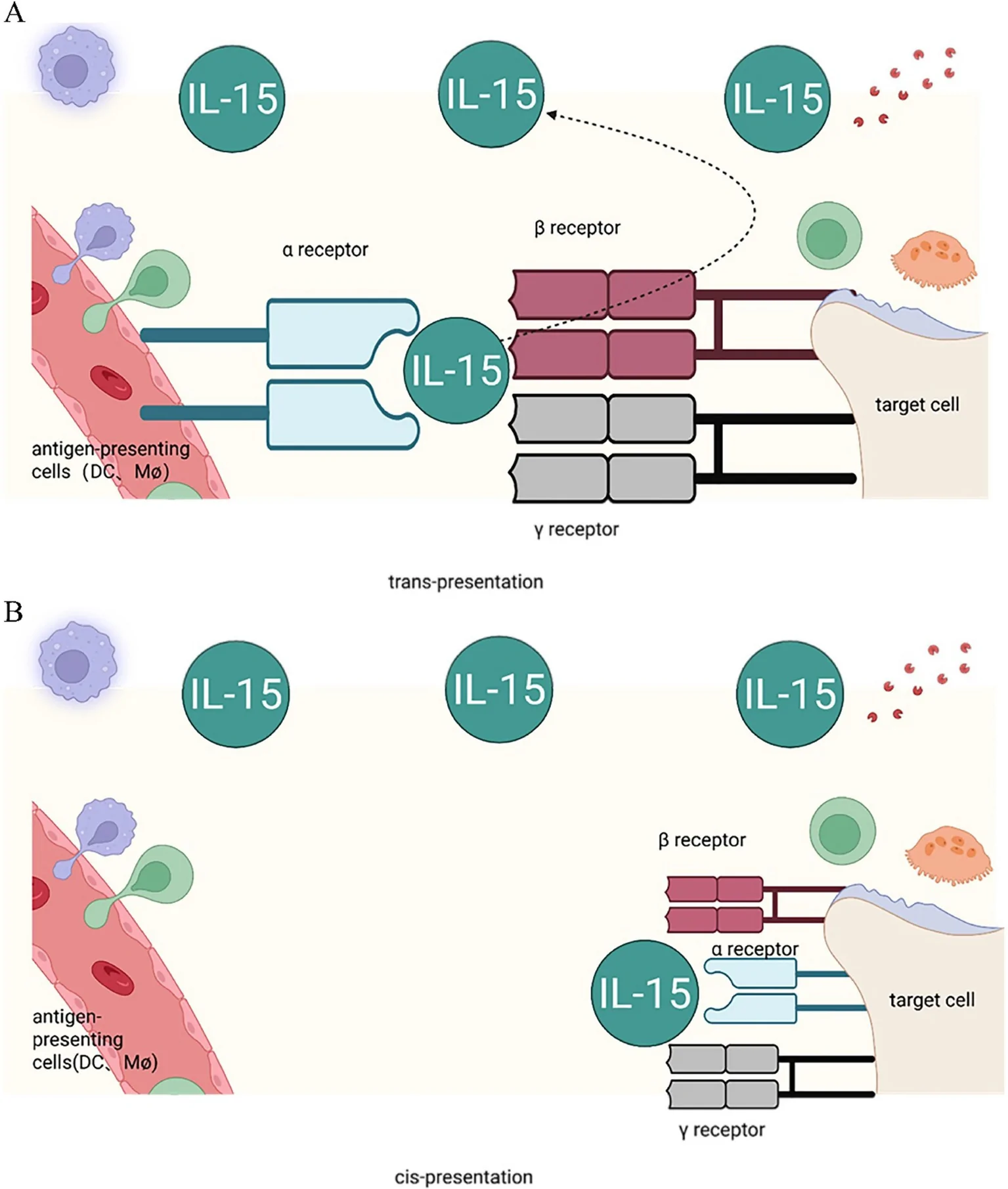
The biological presentation modes of IL-15. **(A)** The trans-presentation of IL-15. The IL-15 receptor a is located on the side of the antigen-presenting cell, and the other two receptors are located on the side of the target immune cell. **(B)** The cis-presentation of IL-15. The three subunits of the IL-15 receptor are located on the same side (i.e., on the side of the target cell). After trans-presentation or cis-presentation, IL-15 regulates the downstream immune response (IL-15, interleukin-15; DC, dendritic cell; Mø, macrophage).

IL-15 has two distinct biological presentation modes including trans-presentation and cis-presentation (37) (Figure 1). Trans-presentation is the most significant and typical action mode of IL-15, and it is also the core characteristic that distinguishes it from IL-2. IL-15Rα has a very high affinity for IL-15, therefore low-dose IL-15 can bind to IL-15Rα. In the trans-presentation mode, low-dose IL15 binds to IL-15Rα on the cell surface (such as dendritic cells, macrophages, hepatocytes and intestinal epithelial cells) to form a ligand-receptor complex, which increases the stability and biological activity of IL-15. Then it is trans-presented to response cells expressing IL-2/15Rβγ (such as resting NK cells and CD8^+^ T cells) to promote the development and homeostasis of memory CD8^+^ T cells, NK cells and iNKT cells, which is the major mechanism mediating IL-15 responses during steady state conditions (38, 39) (Figure 1A). For the cis-presentation mode, it is the formation of ipsilateral polymerization of the three receptors. IL-15 firstly binds to IL-15Rα in either transmembrane form or soluble form, and then binds to IL-15Rβγ of the same cell (Figure 1B). This mode is particularly important for activating NK cells during inflammatory responses and it is likely the dominant mechanism when lymphocytes are treated by recombinant IL-15 *in vitro*. In addition, IL-15Rα is also shed from the membrane, binding to IL-15 and existing in a free form, which can be activated remotely with the IL-15Rβ/*γ* complex. Therefore, IL-15 is possibly an essential factor in controlling the innate and adaptive immune system. The biological effects of IL-15 might be associated with the presentation modes, target cells, receptor structures and concentrations of IL-15, which will be discussed in greater detail below.

### 2.2 Signal transduction pathway of IL-15

**Figure 2:**
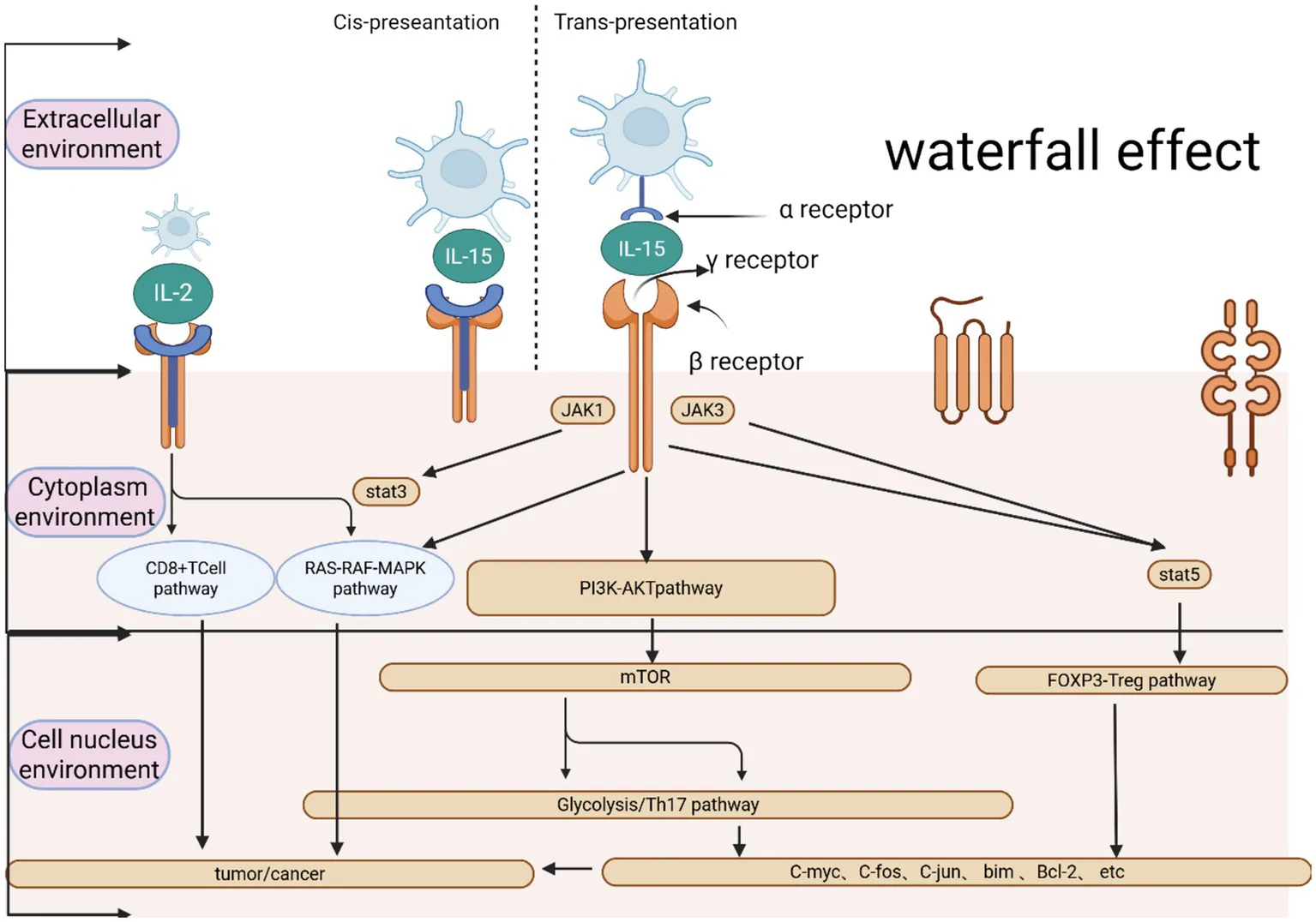
The signal transduction pathway of the trans-presentation of IL-15 and the subsequent biological effects. IL-15 activates the Th17 and Treg pathways through a classic signaling mode similar to IL-2 and its unique trans-presentation mode, and participates in mediating tumorigenesis (IL-15, interleukin-15; JAK, Janus kinase; STAT, signal transducer and activator of transcription; PI3K, phosphoinositide 3-kinase; RAS, rat sarcoma virus; RAF, rapidly accelerated fibrosarcoma; MAPK, mitogen-activated protein kinase; c-myc, cellular myelocytomatosis; c-fos, cellular Finkel-Biskis-Jinkins murine osteosarcoma; Bim, Bcl-2 interacting mediator of cell death; Bcl-2, b-cell lymphoma-2).

The signaling pathway of IL-15 is divided into upstream (cell membrane), midstream (cytoplasm), and downstream (nucleus; Figure 2). Theoretically, on the cell membrane, JAK1 signal is formed by acting on the transmembrane receptor *β* and nuclear transcription factor STAT3 is recruited on the cytoplasmic side; JAK3 signal is formed by acting on the receptor *γ* and STAT5 is recruited. The signal from the outside of the cell membrane is successfully transferred to the inside of the cell. In the middle reaches, phosphorylated STAT not only directly acts on the nucleus, but also helps to activate other signaling pathways. On the one hand it can recruit Shc to the phosphorylation site on the IL-2/15Rβ chain and then activate Grb2, which can phosphorylate Akt along the phosphorylated carnosine 3-kinase (PI3K) pathway. On the other hand, it binds guanine nucleotide exchange factor SOS to activate RAS/RAF and finally activate mitogen-activated protein kinase (MAPK) (40–45). The RAS-MAPK pathway and PI3K-AKT pathway can interact. In the downstream, three main signaling pathways (JAK/STAT pathway, PI3K/AKT/mTOR pathway and MAPK pathway) continue play the effects. The phosphorylated STAT3 and STAT5 proteins form heterodimers, which are directly transferred to the cell nucleus to activate the transcription of anti-apoptotic protein bcl-2 and proto-oncogenes c-myc, c-fos and c-jun. Among them, STAT5 is closely related to IL-15, and STAT5 is a nuclear transcription factor, which plays a role by binding to DNA (46). The activated PI3K-AKT pathway initiates further downstream signaling through mTOR, then induces the expression and activation of downstream effector molecules such as c-myc, c-fos, c-jun, Bcl-2, and NF-kB. The products of MAPK signaling promote cellular proliferation, cytokine production, and antigen-specific expansion in immune cells (47). In short, IL-15Rα is widely expressed in humans and mice and the basic mechanism of IL-15 signaling pathway is that IL-15 stays on the cell surface and forms immune synapse with IL-2R/IL-15Rβ*γ*c on nearby effector NK cells or T cells when IL-15 binds to IL-15Rα subunit on cells (48, 49). Furthermore, the activation of the JAK1/JAK3 and STAT3/STAT5 pathways, Syk kinase and phospholipase C(PLC) γ, Lck kinase and Shc lead to the activation of the PI3K/Akt and Ras/Raf/MAPK signaling cascades. These pathways are the Core driving factors which lead to subsequent expressions of Bcl2, Myc and Fos/Jun and NFkB activations (50, 51). Notably, the different signaling pathway determines the pleiotropic functions of IL-15.

### 2.3 The pleiotropic functions of IL-15

IL-15 has a broad range of biological effects. It has synergistic or antagonistic effects with other cytokines to exert the immune regulatory network of the body as an important substance.

IL-15 is an essential factor of the development of NK cells, T cells and some endothelial cells, it is a growth factor and regulator of memory CD8^+^ T cells, which can induce the activation and induce the proliferation of CD8^+^ T cells and lymphokine activated killer cells (44, 52–55). Firstly, IL-15 induces the proliferation of NK cells to fight viral infection. The cell surface of plasmacytoid dendritic cells (pDC) expresses IL-15R*α*, type 1 IFN-α/*β* induces pDC to produce IL-15, which binds to IL-15Rα to form IL-15/IL-15Rα complex. Then the complex is presented to NK cells in a trans- presentation form to initiate a variety of cellular functions (56). Secondly, IL-15 not only promotes the production of memory CD8^+^ T cells, but also plays a crucial role in maintaining memory CD8^+^ T cells in the body. IL-15, like IL-2, provides early stimulation for T cell activation, but IL-15 can block IL-2-induced apoptosis. IL- 15 supports the persistence of memory CD8^+^ T cells and is important for the maintenance of long-term anti-tumor immunity, and IL-15-related drugs have entered clinical studies in cancer and hematological malignancies (57–59).

**Figure 3:**
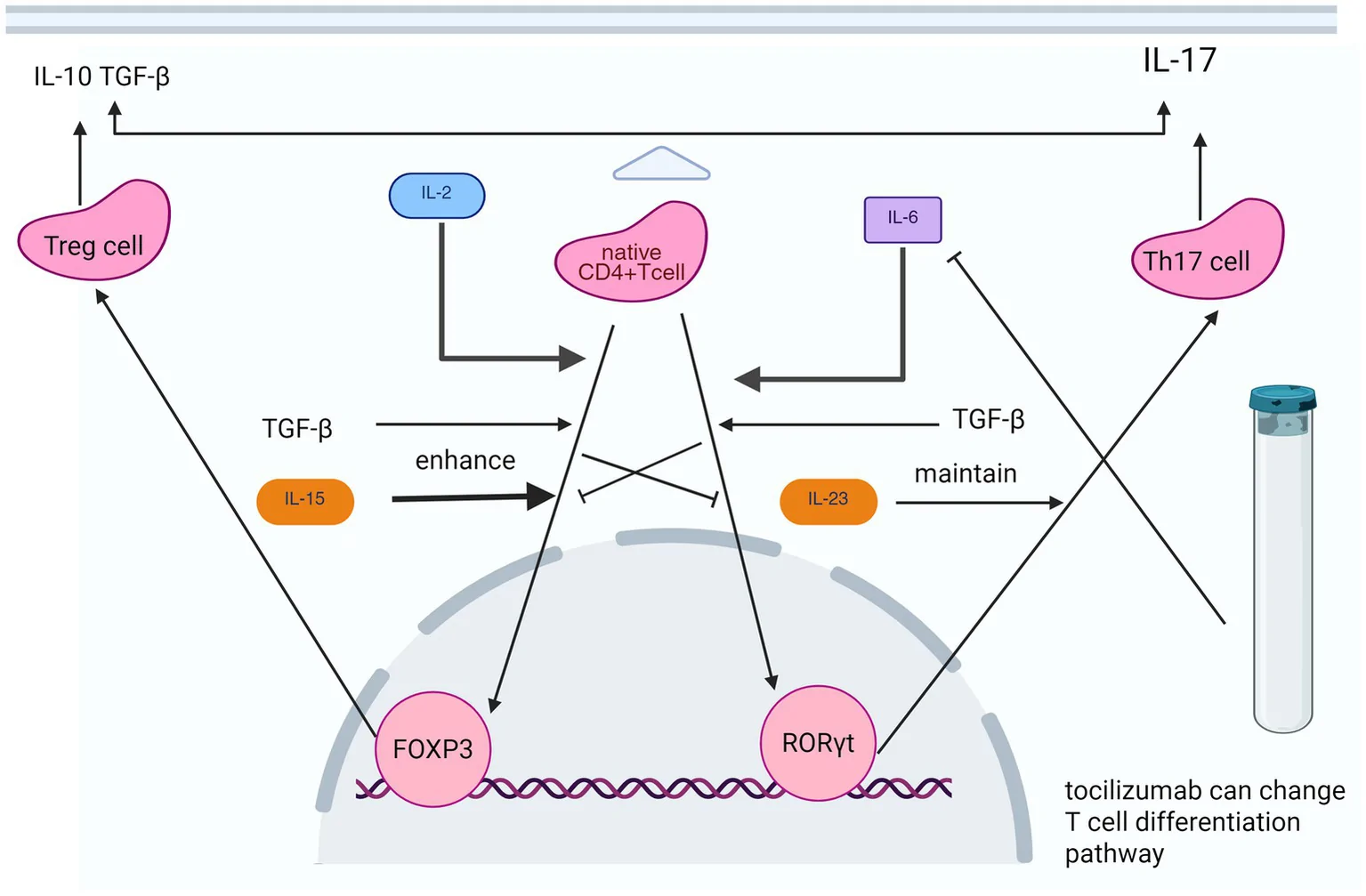
The differentiation of Treg cells. In the absence of IL-2, the presence of TGF-*β* and IL-6 acts on the initial T cells exerts biological effects through the JAK-STAT3 pathway to promote Th17 cells through the RORγt pathway, which plays an immune response role. However, the presence of IL-2 and TGF-β acts on the CD25 receptor to act on the nuclear transcription factor FOXP3 to promote the native T cells differentiate into Treg cells. IL-15 also promotes the differentiation of Treg cells. The two differentiation pathways have reciprocal inhibitory effects, and the IL-6 antagonist Tocilizumab enhances immune tolerance and inhibits the differentiation of Th17 by blocking the signal of IL-6 (IL-15, interleukin-15; IL-10, interleukin-10; IL-2, interleukin-2; IL-6, interleukin-6; IL-17, interleukin-17; IL-23, interleukin-23; TGF-β, transforming growth factor-β; FOXP3, Forkhead box P3; RORγt, Retinoic acid receptor-related orphan receptor gamma t).

It is worth noting that IL-15 also exerts negative regulatory effects. It is well known that low-dose IL-2 activates the proliferation of Treg cells. When TGF-*β* is present, IL-2 promotes FOXP3 by acting on STAT5 then stimulates proliferation and differentiation of Treg cell. While when IL-6 is present, STAT3 activates the specific transcription factor orphan nuclear receptor (RORγt) to express RORγt, which promotes the secretion of IL-17 from Th17 cells. The process of Treg cells is also regulated by IL-15 (Figure 3). When the level of IL-2 is decreased due to various factors such as age, the number, activity and differentiation of Treg cells will decrease or stop, then the activity of Treg cells is maintained by IL-15. A study has confirmed that IL-2 and IL-15 both facilitate the differentiation of thymus-derived regulatory T-cells precursor cells into FOXP3^+^cells while they also promote the proliferation and survival of Treg cells (14). Besides, current studies suggest that when IL-2 is insufficient, IL-15 maintains the activity of Treg cells by inhibiting bim and partially replacing IL-2 (60–62). According to Gene Expression Omnibus (GEO), exogenous IL-15 is sufficient to promote allogeneic maturation (mo) DC-induced the amplification and survival of nTreg cells without the need for IL-2 (63). And in newborns, when there is almost no recirculated Treg in the thymus, the deficiency of IL-2 leads to a significant reduction in Treg development, while the deficiency of IL-15 is significantly more pronounced (18). It has been found that approximately half Treg in the thymus was reduced in IL-15 deficient mice compared to control mice (64). The above indicates that IL-15 is involved in the development of Treg cells.

In addition, the growth-promoting effect of IL-15 is not only reflected in immune cells, but also in other tissues and organs. IL-15 stimulates the proliferation of fibroblast/lipogenic progenitor cells (FAPs) *in vitro* and *in vivo*, and prevents the adipogenesis of FAPs. IL-15 induced FAPs growth is mediated through the JAK–STAT pathway. Moreover, studies have shown that IL-15 can cause fibrosis after muscle injury and promote the regeneration of muscle fibers (65, 66). Finally, the expression of IL-15 was correlated with the severity of fibrosis and the number of FAPs in patients with chronic rotator cuff tears. IL-15 can affect fat formation or reduce fat mass, that is, IL-15 can stimulate the proliferation of FAPs through the JAK–STAT pathway. IL-15 can promote collagen deposition in the body after muscle injury, and this process can be stopped by blocking the JAK–STAT pathway. In addition, some scholars have reported that IL-15 also regulates pancreatic remodeling and thus plays an anti-pancreatic fibrosis role (67), but the opposite role in muscle and pancreas does not necessarily represent the existence of contradiction, that is, there may be tissue specificity. Given that the remodeling process of tissues relies on adequate vascular support to sustain metabolic demands. The combined use of CD34 and CD105 was proven valuable in quantifying active angiogenesis (68). The specific effects of IL-15 in tissues such as muscles or pancreas may also involve some potential angiogenic components, and CD34 and CD105 might serve as potential markers.

The biological effects of IL-15 might be associated with the presentation modes, target cells, receptor structures and concentrations of IL-15. The trans-presentation of IL-15 mainly provides continuous survival and slow proliferation signals for resting NK cells and memory CD8^+^ T cells, without triggering a strong inflammatory response, by the low-dose, contact-dependent approach (69). However, the cis-presentation of IL-15 is particularly important for activating NK cells during inflammatory responses and it is likely the dominant mechanism when lymphocytes are treated by recombinant IL-15 *in vitro*. When IL-15 acts on the immunosuppressive Treg cells, it can induce the expression of FOXP3 to maintain immune tolerance. The different concentration of IL-15 might alter signaling outcomes. Deficiency of IL-15 leads to deficiency in immune cells such as NK and memory CD8^+^T cell. IL-2R and IL-15 share the IL-2/15βγ, high-dose IL-2 and IL-15 activate Th1, Th2 as well as Th17 cells to initiate positive immune effects. The increased IL-15 activated three main signaling pathways to initiate and maintain inflammation and autoimmunity, a robust continuous signaling by IL-15 leads to increase in cytotoxicity in addition to survival, proliferation, metabolism, and cell motility which leads to an imbalance in immune response causing tissue damage. However, the activation in STAT5 pathway is first activated by the IL-2 high-affinity receptor (i.e., the presence of CD25) (70–72), therefore low-doses IL-2 activates the proliferation of Treg cells (9, 73, 74). Similarly, at physiological and moderate levels, the STAT5 in the IL-15/IL-15R signaling pathway is also preferentially activated to maintain immune homeostasis and memory (47). Additionally, the pleiotropic effects of IL-15 also depend on the cytokine network in the microenvironment. The expression of IL-15 is increased by lipopolysaccharide (LPS), type I IFN-*α*/*β*, type II IFN-*γ* and TGF-β, which also induces IL-15Rα and enables receptor-mediated trans-presentation to neighboring lymphocytes. IL-15 is increased under LPS, type I IFN-α/β and type II IFN-γ to exerts pro-inflammatory effects. While TGF-β interacts IL-2 or IL-15 to induces the initial CD4^+^T cells to express FOXP3 and CD25 by acting on the STAT5 pathway, thus forming FOXP3 + CD25 + T cell (active Treg cells). Notably, recent study has found that IL-15 in hair follicles maintain immune privilege (75). In summary, regulating the concentration of IL-15 to promote the shift from immune responses to immune balance is a new direction in the future.

## 3 The pleiotropic immunomodulatory effects of IL-15 in AA pathogenesis

IL-15 is generally regarded as a crucial pathogenic cytokine in AA. However, with the further exploration of the pleiotropic function of IL-15 and the concept of hair follicles as IP sites, IL-15 may exert pleiotropic immunomodulatory effects in the pathogenesis of AA.

### 3.1 Pro-inflammatory effects: IL-15 is a proinflammatory cytokine in AA

Several studies have shown the importance of IL-15 in the survival of memory CD8^+^ T cells. Also, recent studies have shown that IL-15 promotes the production of a new subset of pathologic CD8^+^ memory-related T cells, CD44^super-high(s-hi)^CD49d^lo^CD8^+^ T cells, to participated in AA (76). Because of immune memory is antigen-specific, most CD8^+^ T cells express naive phenotypes (namely naive T cells) in an unmanipulated host, but virtual memory T (TVM) cells are a T cell subtype with a memory phenotype but are unexposed to antigens. The function of these cells differently from naive T cells and are able to induce cytokine production more quickly in response to the stimulation of T cell antigen receptor and cytokine. Compared with naive T cells, TVM cells express higher CXCR3, and its ligands CXCL9 and CXCL10 are up-regulated in AA skin. As a result, TVM cells have the potential to migrate to the skin. A TVM cell-originated CD44^s-hi^CD49d^lo^CD8^+^T cell subset with ultra-high expression of CD44 but low expression of CD49, which not only has the characteristics of tissue retention (such as skin) but also promotes its innate cytotoxicity via natural killer group 2 member D(NKG2D)-dependent mechanism. NKG2D is an effective regulator of NK cell activation, which is expressed on NK cells, certain T cell populations including CD8^+^, NKT and *γ*/*δ* T cell subsets. NKG2D triggers downstream signaling cascades in effector cells to form cytolytic synapses (77–79). CD44^s-hi^CD49d^lo^CD8^+^ T cells were induced from conventional TVM cells by IL-12, IL-15, and IL-18. And IL-2, IL-7 and IL-15 significantly enhanced the proliferation of CD44^s-hi^CD49d^lo^CD8^+^ T cells. In addition, IL-15 stimulation further increased the expression of NKG2D on CD44^s-hi^CD49d^lo^CD8^+^ T cells, which leads to the AA (76). In short, IL-15 plays an important role in promoting AA by inducing tissue-resident pathological CD44^s-hi^CD49d^lo^CD8^+^ T cells. Besides, IL-15 stimulates IFN-*γ* secretion in CD8^+^ T cells through JAK1/3-dependent signaling and establishes a positive feedback loop. Perhaps this cascade undermines hair follicle immune privilege and leads to extensive infiltration of inflammatory cells surrounding the follicle. During the growth period of patients with alopecia areata, the immune immunity of hair follicles is destroyed, and self-reactive CD8^+^ T cells produce IFN-*γ*, which binds to IFN-γ receptors of hair follicle epithelial cells and induces IL-15 production through the JAK1/2-STAT pathway (80). IL-15 binds to receptors on CD8^+^ T cells and sends out more signals to stimulate IFN-γ production via the JAK1/3-STAT pathway. This positive feedback loop enhances the inflammatory response, leading to a disruption of the hair growth period leading to hair loss (81).

### 3.2 Anti-inflammatory effects: IL-15 is the guardian of IP in hair follicles

**Figure 4:**
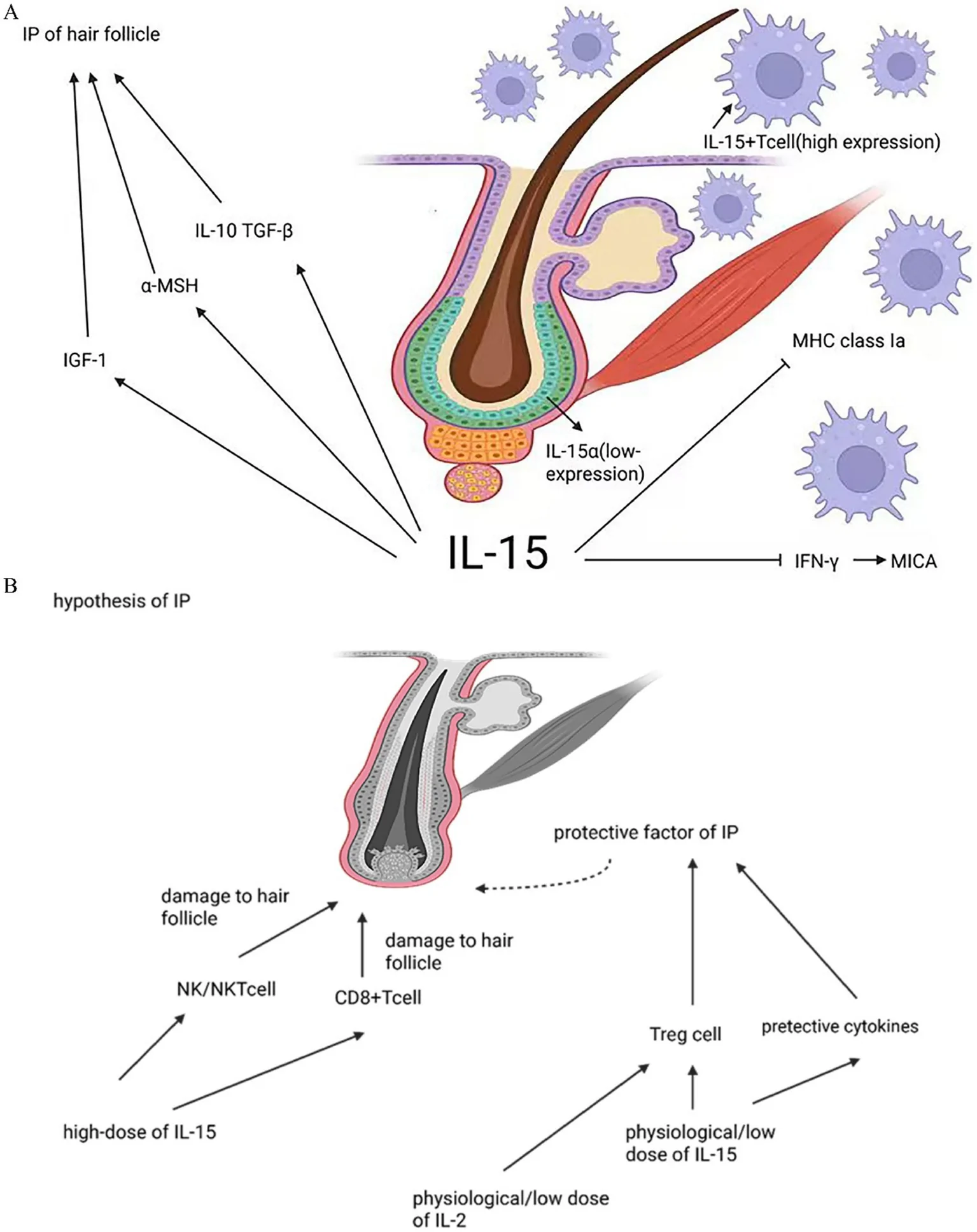
The role of IP in hair follicle. **(A)** The anti-inflammatory effects of IL-15 in regulating IP in hair follicle by promoting TGF-β, α-MSH, and IGF-1 but inhibiting MHC class Ia and IFN-γ. **(B)** The hypothesis of IL-15 in regulating IP in hair follicle under different concentration (IL-15, interleukin-15; IP, immune privilege; α-MSH, α-melanocyte-stimulating hormone; IGF-1, insulin-like growth factor 1; IFN-γ, interferon-gamma; NK, natural killer cell; NKT, natural killer T cells).

In addition to classical proinflammatory effects, recent studies have reported that IL-15 may play an important anti-inflammatory role in AA. Moreover, IL-15-induced signaling effects may also differ between immune cells and hair follicle epithelial cells, which is because that IL-15 may well exert functions that differ from its activities elsewhere in IP-dominated tissue. Recent studies proposed a new view that IL-15 is the guardian of immune immunity in hair follicles (75, 82). They found that 50–100 ng/mL of recombinant human IL-15 (rhIL-15) treatment for 6 days significantly prolonged anagen/inhibited catagen in hair follicles compared with controls and increased percentage of proliferative Ki-67 + keratinocytes in the anagen hair, which significantly promoted hair follicles growth in human scalp, prolonged the growth period, and reduced the apoptosis of isolated hair matrix (HM) keratinocytes (82). They also found that perifollicular IL-15^+^ cells were increased in lesional and non-lesional hair follicles of AA patients, but intrafollicular IL-15, IL-15Rα and IL-15R*γ* protein were decreased in the epithelium of hair follicles in AA patients. Then they explored whether intrafollicular IL-15/IL-15R*α* signaling was essential to maintain IP in hair follicles by adding rhIL-15 in organ-cultured healthy human scalp hair follicles. The result showed that different from the effect of IFN-γ to induce human hair follicle immune immunity breakdown, premature degeneration, and malnutrition both *in vivo* and *in vitro*, rhIL-15 significantly up-regulated *α*-melanocyte-stimulating hormone (α-MSH) protein in HM and hair bulb ORS keratinocytes, which is potently immunoinhibitory IP guardian. On the contrary, rhIL-15 down-regulated major histocompatibility complex (MHC) class Ia in HM, but major histocompatibility complex-class-I-related chain A (MICA) and insulin-like growth factor-1 (IGF-1) were not changed significantly. In short, stimulation with IL-15 may strengthen IP function of hair follicle, rather than weakening it (75). IL-10-secreting inhibitory natural killer T cells (iNKT10) has been confirmed to promote hair regrowth in AA lesions *in vivo* (83). They also found that rhIL-15 restored both the number and the percentage of iNKT10 + cells among the total number of iNKT cells in the papillary dermis by studying rhIL-15 impacts on iNKT10 cells in healthy organ-cultured, full-thickness human eyelid skin. Therefore, IL-15 also indirectly protected IP in hair follicles by promoting the survival and IL-10-secretory activity of AA-protective perifollicular immune cells. And they also found that rhIL-15 treatment could stimulated hair regrowth *in vivo* rather than promoting AA-associated hair loss (75). Taken together, IL-15 exerts the anti-inflammatory effects to maintain IP in hair follicles through many approaches (Figure 4A), and it also can promote hair regrowth in established AA lesions, which encouraged IL-15R agonists as a therapeutic intervention for hair growth of AA therapy. In conclusion, the expression of IL-15, IL-15Rα, and IL-15Rγ proteins in the epithelial cells of alopecia areata hair follicles was decreased, and the composition of the IL-15/IL-15R signaling environment in the hair follicles was impaired. Although it is not clear whether this represents a structural defect in patients with alopecia areata, or whether this phenomenon is related to the presence and increased infiltration of IL-15^+^ cells around the follicles, but it can be confirmed that IL-15 is not necessarily just a substance that amplifies the maintenance of the immune response. To a certain extent, IL-15 also plays a role in promoting tissue regeneration and restoration of immune privileges. Therefore, selective stimulation of IL-15Rα signaling in hair follicles may be a new treatment for AA while pharmacologically blocking it may hinder HFs-IP recovery and hair regrowth, even potentially making hair follicles more susceptible to recurrence of alopecia areata AA.

The function of Treg cells is mainly to maintain autoimmune tolerance and as an opposite regulatory inflammatory factor to prevent the occurrence of chronic inflammation. The differentiation process of Treg cells is regulated by multiple cytokines including IL-15 (Figure 3). Studies have confirmed that the inhibitory activity of circulating Treg cells from AA patients was impaired (84). Tissue-resident Treg cells, a special population of Treg cells, are distributed in specific peripheral tissues to maintain tissue homeostasis and wound repair (85). Skin is the largest organ and is home to a large proportion of the body’s tissue-resident Treg cells, namely skin-resident Treg cells. It preferentially localizes in hair follicles as IP guardians to promote wound healing, tolerance and drive the cyclic growth of hair follicles (86). In the lesional scalp of AA, skin-resident Treg cells in the follicles are impaired, leading to dysregulated local immunity and hair follicle regeneration disorders such as AA (22, 87). The Treg-based therapies for AA might be potential because the induction of Foxp3 + Treg cells and activation of skin-resident Treg cells helps re-establish immune homeostasis and promote HF regeneration in the lesional scalp (22). Low-dose IL-2 is an effective therapy for autoimmune diseases by inducing Treg cells, and some promising data also have been achieved with IL-2 to treat AA (9–11). IL-15 also promotes the expression of Treg cells (14, 60–63). However, it is still unclear whether IL-15 promotes the expression of skin-resident Treg cells, thereby facilitating hair follicle regeneration. Based on the multi-effect immune effects of IL-15, we hypothesize that at physiological and low-dose IL-15 preferentially maintains IP in hair follicle by increasing Treg cells, however, direct evidence in AA remains lacking (Figure 4B).

In summary, IL-15 exerts a dual role in the pathogenesis of AA. On one hand, high doses of IL-15 promote the expression of NK cells and CD8 + T cells to cause the inflammatory damage and impair IP in the hair follicles. On the other hand, physiological and low-dose IL-15 maintains the IP of hair follicles by directly or promoting the expression of Treg cells to exert a negative regulatory effect (Figure 4B). However, the available evidence includes human AA tissue analyses, ex vivo human hair follicle and mouse models (Table 1). The role of IL-15 in human hair follicles is best supported by evidence directly from human AA tissues and hair follicles (75, 82). Future studies should integrate dose–response analyses with spatial and single-cell approaches to determine how IL-15 concentration, cellular source, mode of presentation, and local inflammatory context jointly determine its biological effects in human AA tissues.

**Table 1:** The comparison of IL-15 effect in different studies of AA.

Study	Model	IL-15 exposure	Main finding	Key limitation	Relevance to human AA
Suzuki et al., 2024 (76)	Human AA tissue; ex vivo human hair follicle; human hair follicle organ culture	rhIL-15 exact concentration reported in original study	IL-15 exerts the anti-inflammatory effects to maintain IP in hair follicles, and promotes hair regrowth in established AA lesions	Ex vivo model; limited clinical validation	High
Suzuki et al., 2024 (82)	Human AA tissue	rhIL-15 (1, 50 and 100 ng/ml)	IL-15 promotes hair follicles growth in human scalp	Ex vivo model; limited clinical validation; Organ culture	High
Seok et al., 2023 (75)	AA mouse model; cell culture	IL-15	IL-15 promotes the production of a new subset of pathologic CD8^+^ memory-related T cells, CD44^super-high(s-hi)^CD49d^lo^CD8^+^ T cells, to participated in AA	Human AA tissue; ex vivo human hair follicle	Moderate

## 4 IL-15-based therapies: opportunities or challenges?

### 4.1 Previous perspectives of treatment strategies for alopecia areata and its limitation

The occurrence and development of alopecia areata are often related to the overactivation of JAK–STAT pathway, so biologics can be used to treat alopecia areata when drugs such as hormones are not effective. In clinical practice, with few treatment options currently available for severe AA. To date, the FDA approved systemic agents for the treatment of severe AA including the Janus isozyme (JAK) 1/2 inhibitor Baricitinib, JAK1 inhibitor Upadacitinib and the selective JAK3/tyrosinase inhibitor Ritlecitinib. None of these treatments reliably suppress disease recurrence. This may be due to the fact that these treatment regimens mainly focus on anti-inflammatory effects, while neglecting the pleiotropic effects of cytokines and the complex JAK–STAT downstream signaling network. The latest research has found that IFNγ+ NK cells drive the homeostasis expression of TRAIL in astrocytes, and NK cells exhibit anti-inflammatory functions, which is related to the reduction of disease severity in MS (multiple sclerosis) patients. And IL-2 signal modulators normalize the function of NK cells in MS patients. Moreover, IFNγ-driven TRAIL+ astrocytes restrict central nervous system inflammation by inducing T-cell apoptosis through Trail-DR5 signaling (88, 89). Therefore, the inflammatory effects and immune imbalance in the pathogenesis of alopecia areata should be taken seriously.

### 4.2 New directions of future treatment strategies for alopecia areata and its innovativeness

In AA, higher serum levels of IL-2 and IL-12 may be critical, as they contribute significantly to the activation of CD8^+^ T cells into effector cells. Although some data showed that low-dose IL-2 had promising perspective in treating AA by inducing Treg cells (9–11), some studies also indicated that there was no significant positive improvement in both C3H/HeJ mouse AA models and placebo-controlled trials in patients with moderate to severe AA under the use of low dose IL-2, which leads to questions about the therapeutic benefits derived from Treg-mediated immunosuppression with the use of low dose IL-2 (90). As already mentioned, In alopecia areata, IL-15, a member of the IL-2 family, has increased levels around the hair follicle and significantly decreased levels inside the hair follicle, suggesting a potential role of IL-15 in maintaining normal human hair growth and immune tolerance (75). Furthermore, early studies have identified a critical role for IL-15 in maintaining tissue-resident Treg cells within the skin. Last but not least, when IL-15 co-cultured with dermal fibroblasts, skin-resident Treg cells will proliferate in an antigen-independent manner (91), which expanding Treg cells to suppress local inflammatory responses, further emphasizing the potential therapeutic value of IL-15 in AA.

Although temporarily direct application of IL-15 in clinical practice in AA is still in the exploratory stage, there are many cell experiments and animal experiments have confirmed that IL-15 has the effect of regulating immune tolerance and even directly regulating the function of Treg cells (Table 2). As early as 2006, human cell experiments proved that IL-15 has anti-inflammatory effects, and this effect was confirmed in clinical trials in the same year (92, 93). In the following 10 years, there were successive reports on IL-15 as an anti-inflammatory factor. For example, IL-15 promotes regulatory T cell function and protects against diabetes development in NK-depleted NOD mice in 2010, IL-15-dependent balance between Foxp3 and RORγt expression impacts inflammatory bowel disease in 2016, and IL-15 is a hair follicle immune privilege guardian in 2024, and so on (15, 75, 94–99). More and more evidence points to the potential anti-inflammatory effect of IL-15. In 2025, a study shows that rhIL-15 and IL-15 hyper agonists can alleviate skin inflammation in patients with psoriatic lesions (100). Therefore, IL-15 is a pleiotropic cytokine, IL-15 may be different biological effects through different JAK–STAT pathway signaling pathway, IL-15 in alopecia areata has the immune tolerance mechanistic insights and necessary translational perspectives.

**Table 2:** Key human studies, animal experiments and cell experiments about IL-15.

Year	Type	Direction	Description	Document number
2006	Organ culture of human colonic explants	Anti-inflammatory role of IL-15 in Crohn’s disease	It is suggested for the first time that IL-15 has potential anti-inflammatory effects. Th1 stimulatory effect was inhibited by IL-15 in a dose-dependent fashion.	(92)
2006	In two different experimental models of colitis in vivo, and in models of intestinal epithelial cell (IEC) apoptosis *in vitro*.	IL-15 protects intestinal epithelial cells	IL-15 not only acts as a pro-inflammatory factor, but also has potential anti-inflammatory effects. Endogenously produced IL-15 in chronic colitis does not only act as a proinflammatory cytokine but has at the same time the potential to reduce mucosal damage by preventing IEC apoptosis.	(93)
2010	Mice experiment: diabetes in NOD mice.	IL-15 promotes regulatory T cell function and protects against diabetes development in NK-depleted NOD mice	IL-15 has the effect of promoting Treg function and preventing diabetes in NOD mice, but such an activity can be eliminated by simultaneous activation of NK cells in IL-15-treated mice.	(94)
2014	Cell experiment: IL-15-CFP knock-in mice	Characterization of the IL-15 niche in primary and secondary lymphoid organs in vivo	In spleen, the expression of IL-15 is age-dependent.	(95)
2016	Mice experiment	IL-15-dependent balance between Foxp3 and RORγt expression impacts inflammatory bowel disease	Impaired delivery of IL-15 to CD4 + T cells in the colon downmodulates Foxp3 expression (diminishing STAT5 phosphorylation) and enhances RORγt expression (by upregulating the expression of Runx1). IL-15 can impair the upregulation of RORγt and IL-17 expression and impaired delivery of IL-15 to CD4 + T cells can lead to IBD.	(96)
2018	Important review	IL-2 and IL-15 dependent thymic development of Foxp3-expressing regulatory T lymphocytes	It is tempting to speculate that, with age, Treg differentiation will decreasingly depend on IL-2-producing dendritic cells and progressively on interactions with IL-15-producing mTEC. With the increase of age, IL-2 will gradually decrease and the body will gradually rely on IL-15.	(15)
2019	Cell experiment:	Comparison of IL-2 vs. IL-7/IL-15 for the generation of NY-ESO-1-specific T cells	IL-7/IL-15 led to an increase of CD4^+^ T cells and a decrease of CD8^+^ T cells, enriched the amount of naïve-like T cell among CD4^+^ T cells but not among CD8^+^ T cells.	(97)
2020	Cell experiment: naïve CD4^+^ T-cells from a human donor	Investigation of the impact fromIL-2、IL-7 and IL-15 on the growth and signaling of activated CD4 + T cells	The most favorable conditions for T cell growth study, and from the predictive model align to include 10 ng/mL for IL-2 and 36 ng/mL for IL-15, followed by 36 ng/mL for IL-7 and 36 ng/mL for IL-15, followed by 10 ng/mL of IL-2 and 36 ng/mL for IL-15.	(98)
2023	A lymphocytic choriomeningitis virus (LCMV) Armstrong infection mouse mode	Regulatory T cell-derived IL-15 promotes the diversity of immunological memory	Treg cell-derived interleukin (IL)-15 is required for long-term maintenance of the KLRG1^+^ IL-7Rα^−^ CD62L^−^ terminal effector memory CD8^+^ T (tTEM) cell subset.	(99)
2024	Human autoimmunity of AA patients and experimental AA induction in vivo	Interleukin-15 is a hair follicle immune privilege guardian	Quantitative immunohistomorphometry showed the number of perifollicular IL-15 + T cells in AA skin biopsies to be significantly increased compared to healthy control skin, while IL-15, IL-15Rα, and IL-15Rγ protein expression within the hair bulb were significantly down-regulated in AA HFs.	(75)

### 4.3 Safety considerations and risk-mitigation strategies for IL-15-based interventions

Although IL-15 may contribute to IP in hair follicle, its therapeutic application requires careful consideration of its pleiotropic and potent immunostimulatory effects. Given IL-15 is a key regulator of lymphoid homeostasis and promotes the survival, proliferation, and functional maintenance of NK cells and memory CD8^+^ T cells. The sustained or dysregulated IL-15 signaling may result in excessive immune-cell activation or expansion. And IL-15 dysregulation is also associated with multiple autoimmune and inflammatory diseases (27, 101). The effects of IL-15 on tissue remodeling and fibrosis appear to be context dependent. IL-15/IL-15Rα signaling has been implicated in liver fibrogenesis, whereas IL-15 has shown anti-fibrotic effects in experimental models of renal fibrosis and chronic pancreatitis (67, 102, 103). Whether IL-15 influences perifollicular fibrosis or extracellular matrix remodeling in AA remains unknown. Long-term preclinical studies should therefore evaluate potential changes in hair follicle architecture, collagen deposition, and tissue remodeling. To improve the therapeutic index of IL-15-based interventions, future approaches should control IL-15 signaling and use the local or topical delivery to reduce systemic exposure, which may limit sustained activation of NK cells and cytotoxic CD8^+^ T cells. Engineered or conditionally active IL-15-based agents may further enable control of cytokine distribution, exposure duration, and receptor engagement. However, these approaches remain to be validated in AA.

## 5 Summary

The occurrence and development of AA is due to chronic autoimmune inflammation in hair follicles mediated by impaired IP. In the past, IL-2, 15 was generally considered to positively regulate immunity by regulating the proliferation of Treg and promoting growth and repair to a certain extent. IL-15 has been suggested in recent studies to promote the repair of hair follicles and the production of immune tolerance cytokines, which have similar effects and possible links with low dose of IL-2. When the level of IL-2 in the body decreases due to various factors such as aging, the number and activity of Treg cells and their differentiation will decrease or stagnate. At this time, Treg cells in the body will rely more on the action of IL-15 to maintain the activity of Treg cells. Given that IL-15 plays a dual role in the pathogenesis of AA, how to inhibit its pro-inflammatory effects but enhance its anti-inflammatory effects will become a promising direction for the treatment of AA with IL-15, which needs further exploration in the future.

## 6 Limitations and future outlook

There are some limitations in our review. First, the available findings are derived from heterogeneous experimental systems including *in vitro* cell-based studies, animal models and ex vivo human tissues, which may limit the direct comparability and generalizability of these results. Thus, future studies should employ standardized and disease-relevant models to avoid heterogeneous models. Second, there were rare direct mechanistic evidence from human AA tissues, the integrative approaches combining single-cell and spatial transcriptomics, multiplex imaging and functional ex vivo studies in human AA tissues are required to define these mechanisms. Third, the proposed concentration-dependent effects of IL-15 is a hypothesis rather than an established mechanism. Future studies should systematically evaluate dose–response relationships across relevant cell populations and disease states and determine whether the proposed protective and pro-inflammatory effects can be attributed to IL-15 concentration itself. Fourth, whether IL-15 indirectly promotes the regeneration of hair follicle by regulating angiogenesis around the hair follicles remains unknown. CD34 and CD105, as tools for evaluating angiogenesis, might be used to assess whether the IL-15 signal regulation is accompanied by the activation of the surrounding endothelial cells or the formation of new blood vessels through the double immunofluorescence staining of CD34/CD105. Introducing this methodological framework into the research field of AA may provide a new perspective for observing the tissue remodeling effect of IL-15. Finally, because of the critical role of Treg cells in restoring immune tolerance and facilitating tissue repair, therapeutic strategies targeting Treg cells could represents a revolutionary approach for treating a new spectrum of immune-mediated disorders, including AA. The role of IL-2 and IL-15 in peripheral Treg recycling is a new area for future research in alopecia areata and other autoimmune diseases. The association of IL-15 with skin-resident Tregs in hair follicle is worth studying in the future. Addressing these limitations is essential for establishing the biological relevance and translational potential of IL-15-based strategies in AA.
